# Pregabalin abuse and dependence during insomnia and protocol for short-term withdrawal management with diazepam: examples from case reports

**DOI:** 10.5935/1984-0063.20200129

**Published:** 2021

**Authors:** Basavaraja Papanna, Carlo Lazzari, Kapil Kulkarni, Sivasankar Perumal, Abdul Nusair

**Affiliations:** 1 Essex Partnership University NHS Foundation Trust, Psychiatry - Colchester - Essex - United Kingdom.; 2 South-West Yorkshire NHS Trust, Psychiatric Intensive Care Unit - Wakefield - South Yorkshire - United Kingdom.

**Keywords:** Pregabalin, Sleep, Substance Withdrawal Syndrome, Benzodiazepines, Diazepam

## Abstract

**Introduction:**

Pregabalin (PGN) is an anxiolytic, analgesic, antiepileptic, and hypnotic medication. There are concerns about its abuse in the community for managing chronic insomnia and other risks when assumed in overdose or combination with other abuse substances. PGN is classified as a controlled medication. While its discontinuation is accompanied by rebound insomnia and other neurological symptoms, cross-tapering PGN with short-term diazepam (DZ) during inpatient admissions has shown promising results in dealing with PGN withdrawal symptoms accompanied by rebound insomnia.

**Material and Methods:**

We report three cases that began abusing their prescribed PGN. During hospital admission, our teams used a protocol for cross-tapering PGN with DZ to reduce withdrawal symptoms. Other sedative medications are suspended while alcohol is not allowed if patients are on leave from the hospital. Standardized scales for assessment were clinical global impression scale-severity (CGI-S), generalized anxiety disorder scale (GAD-7), and insomnia severity index (ISI).

**Results:**

The cross-tapering PGN with DZ showed similar clinical outcomes with reduced withdrawal symptoms and rebound insomnia during two weeks of cross-tapering. Eventually, DZ, too, is stopped in the hospital to avoid another dependence syndrome.

**Conclusion:**

As emerging in the current study, PGN has strong addictive effects in people who have insomnia and is mostly abused for its hypnotic or sleep-inducing properties when other medications have failed. As applied in the current study, DZ can manage PGN withdrawal symptoms with rebound insomnia while cross-tapering. DZ is then discontinued.

## Introduction

The International Classification of Sleep Disorders (ICSD-III) grades insomnia as: 1) *mild:* where a person laments an inadequate amount of sleep or not feeling restored after habitual sleep, accompanied by agitation, irritability, mild anxiety, fatigue, and tiredness; 2) *moderate*: where the person laments an inadequate amount of sleep or not feeling rested, mild to severe social or occupational working disorder, feelings of restlessness, irritability, nausea, daytime exhaustion, and tiredness; and 3) *severe*: where a person laments an insufficient amount of sleep or not feeling restored after habitual sleep, accompanied by severe social or occupational working disorder and by feelings of restlessness, irritability, anxiety, daytime fatigue, and tiredness^1^.

It is reported that 30% of the general population suffers from insomnia, with daytime after-effects accounting for 9-15%^2^. Insomnia condition affects the standard of living and is correlated with an elevated risk of stress, depression, substance, and alcohol misuse^3^. There are recent reports on the use of pregabalin (PGN) to address treatment-resistant insomnia^4,5^. Pregabalin has been found related to the improvement of quality of sleep when linked to generalized anxiety^6^. PGN enhances the quality of life of those who have insomnia resistant to other hypnotic medication^6^. PGN has been found beneficial in doses of 300mg/daily in controlling insomnia in persons with controlled partial seizures^7^. PGN is also used to treat insomnia in subjects who are dependent on hypnotic benzodiazepines^8^. PGN is prescribed to treat alcohol and benzodiazepine withdrawal syndrome^9,10^. PGN improves sleep quality in users and reduces insomnia in those who have fibromyalgia, neuralgia, generalized anxiety disorder, bipolar disorder, and focal and generalized epilepsy^11-14^. When participants were taking PGN, there was a substantial rise in slow-wave sleep rate and a decline in stage 1 sleep in patients with epilepsy^15^.

However, no matter the clinical use, PGN can become a drug of abuse that needs treatment with benzodiazepines^16^. A systematic review revealed abuse of gabapentinoid (Gabapentin, PGN) of 1.6% in the general population raised to 3% to 68% among abusers of opioid with an increase of more than 75%, since 2012^17^. Nonetheless, PGN can trigger dependence, especially in people trying to find extra help for treating their insomnia; in this case, a medical team might temporarily and for a short-term use benzodiazepines (BDZ) characterized by a long half-life like diazepam (DZ) to combat symptoms of withdrawal from PGN^18^. More problematic is the long-term use of DZ and benzodiazepines in insomnia, especially in older people, those with severe insomnia, and severe anxiety and depression^19^.

PGN withdrawal symptoms include several neuropsychological and rebound signs such as returning insomnia, copious sweating, tachycardia, hypertension, shakes, diarrhea, tension, paranoia, and auditory hallucinations, mutism, self-mutilation, suicidal ideation, headaches, and seizures^20,21^. Multiple research reports that discontinuation of PGN induces withdrawal symptoms comparable to the withdrawal of BDZ, including sleeplessness, severe anxiety, tremor, and restlessness^20^. There are several cases when patients reliant on PGN took it in overdose, planning to die^22^.

In the last five years, there has been a threefold increase in PGN prescriptions in the United Kingdom (UK)^23^. Primary abusers of PGN are patients with a history of polysubstance misuse and those with borderline and antisocial personality disorder; they might present to their family doctor or community psychiatrists intensifying existing symptoms of chronic insomnia or anxiety to have PGN prescribed or increased in its dosage^24^. However, healthcare services in the United Kingdom are recording an increase in deaths due to abuse of PGN, especially in those who want to boost the effects of other substances of abuse such as opioids and alcohol^25-27^. PGN is likely to induce addiction by increasing gamma amino butyric acid (GABA) in the extracellular space of brain neurons; this action is linked to subjective feelings of relaxation and elation at the beginning of PGN use or when assumed in excessive dosage^27^.

Since April 2019, PGN is classified as a controlled drug under the UK Drug Misuse act 1971^28^. PGN has a half-life of 5.5-6.7 hours, independently of dose and repeated dose administration^29^. There is a worldwide concern about PGN abuse leading to national regulations in the UK in prescribing PGN, now classified as a class C controlled substance, after increasing deaths due to its abuse^22,26,27,30,31^. A German study found that multiple-substance abuse was present in 42% of cases in a sample of 55 cases of PGN addiction, with 24% of the cases having a psychiatric diagnosis^32^. An Australian case study found an increase in depression and suicidal ideation in five patients started on PGN^33^.

Therapeutic dosages of PGN in the British National Formulary go from 150 to 600mg daily, while diazepam in a clinical setting is provided to a maximum dosage of 30mg daily^34^. Diazepam, and its metabolite, desmethyldiazepam, have the most prolonged half-life than other benzodiazepines, which means that their plasma level decreases in a self-tapering manner while reducing the risk of seizure by a low degree of rebound phenomena^35^. Diazepam has been one of the most effective treatments since the advent of the psychopharmacological movement that started in the 1950s; it is effective in alleviating the symptoms of CNS conditions, such as anxiety and epilepsy, and it sets the pharmacotherapy benchmark in terms of efficacy, the onset of action, and reliability^36^. Switching and cross-tapering psychotropic medication is a common pharmacotherapeutic intervention in psychiatry^37^. Benzodiazepines, although addictive themselves, are used for a short-term treatment in psychiatric hospitals and by substance misuse teams under close supervision to address withdrawal symptoms from addictive substances, such as vegetative symptoms, anxiety, or risk of seizures from dependence on other substances, such as alcohol or amphetamines^38,39^.

When PGN is discontinued and withdrawal symptoms corrected, occasionally mild insomnia can remain a concern. In this case, sleep hygiene for insomnia remains the most popular form in psychiatric wards due to the protocol’s easiness. Sleep hygiene is mostly based on avoiding caffeine, nicotine, alcohol, promoting physical exercise, reducing stress, decreasing environmental noises in the bedroom, and using regular sleeping^40^. Mindfulness techniques have been reported to improve sleep when coupled with sleep medication^41^. Cognitive behavioural therapy for insomnia (CBTI) has proven to be effective in patients with comorbid depression and insomnia while yielding positive results on other symptoms of depression^42^. The European Sleep Research Society confirms that CBTI is the first-line action for enduring insomnia and that benzodiazepines and some antidepressants are useful for no longer than four weeks^43^. Another thread of research is about the impact of blue light on sleep. It could help patients who have insomnia block short-wavelength blue light in the night hours deriving from computer monitors, laptop screens, tablet computer screens, and smartphone screens^44,45^. Due to light exposure and/or excessive harmful use, blue light-emitting devices (smartphones, tablets, and laptops) at bedtime have detrimental effects on sleep^46^. Blue-blocking (BB) spectacle lenses are advertised to ease eyestrain and trauma by using digital tools, improve the quality of sleep, and theoretically confer safety against retinal phototoxicity by attenuating short-wavelength radiation^47^.

In people with chronic insomnia, PGN has become a popular (self-administered) drug to combat it while, at the same time, creating a dependence syndrome. The challenge of sleep clinicians, and the aim of the current study, is to explore how teams can address insomnia in PGN-addicted patients during hospital admissions, rapidly discontinue PGN, utilize short-term medication to avoid withdrawal symptoms and neurological risks in a controlled environment, and discharge a patient from hospital or services with healthier treatments for insomnia.

## MATERIAL AND METHODS

We report on three cases that characterized the prototypical patients presenting with insomnia and PGN addiction, initially cross tapering with DZ and then switching to non-addictive medication and other sleep aids. All subjects were treated in a psychiatric hospital and were service users under our community psychiatric team’s care. The current research also complies with case report studies by merging a categorical and dimensional approach to diagnosis. Expert psychiatrists collected the data during structured and semi-structured interviews complemented by standardized psychiatric tests. These tests were the GAD-7 (generalized anxiety disorder scale)^48^, CGI-S (clinical global impression scale-severity)^49^, and ISI (insomnia severity index)^50^. Case-study research of a small number of cases or Small-*C* addresses a spatially and temporally delimited phenomenon of theoretical significance^51^. All patients underwent regular physical check-ups at admission, inclusive of hematinic and ECG. An assessment of alcohol use served to rule out severe dependence and intoxication. Insomnia is highly predominant in alcohol use dependence (AUD)^52^. Our clinical team has developed a pharmacological treatment protocol for discontinuing PGN abuse with a short-term and maximum two-week use of DZ. To deal with the risk of seizures at stopping PGN, it was progressively reduced in dosage while DZ managed possible rebound insomnia and withdrawal symptoms in an inpatient regimen. DZ was then stopped before discharge, as the policy is not to prescribe benzodiazepines to non-hospitalized patients due to the risk of addiction and their street values.

## RESULTS

### Case 1

Miss A is a 30-year-old woman with a psychiatric diagnosis of borderline personality disorder (ICD-10: F 60.1) and polysubstance misuse (ICD-10: F 19.0) already followed jointly by our community psychiatric team and substance misuse team. No AUD was elicited from history but only occasional social drinking. She was maintained on regular trazodone 50mg daily. As she claimed to suffer from severe insomnia and anxiety, she started hoarding and abusing PGN to help her sleep. She managed to receive additional amounts of PGN out of the counter from the Internet, piling new prescriptions or drug dealers up to a total of 900mg daily. This way, she exceeded the PGN therapeutic dosage about ten times using it and other abuse substances. When Miss A tried to discontinue PGN by herself, she experienced severe withdrawal symptoms, including untreatable insomnia, anxiety, irritability, anger, and suicidality.

At hospital admission and on standardized scales, she was severely anxious (GAD-7=21); at ISI, she scored 26, indicating clinical insomnia, at CGI-S, she scored six as extremely ill. Her PGN withdrawal symptoms were managed with endpoint regular diazepam at 2mg twice daily, which was finally discontinued before discharge. After two weeks, there was a noticeable improvement in her sleep problems along with the reduction of the level of anxiety (GAD-7=5: mild anxiety), insomnia (ISI=14: subthreshold insomnia), and severity of illness (CGI-S=2: borderline mental illness). She was also referred to a clinical psychologist to start a course of CBTI and mindfulness-based therapy (MBT) for insomnia together with a sleep-hygiene (SH) protocol (CBTI-MBT-SH) while the community psychiatry team continued the follow-up. No other sleep medication was prescribed at discharge. At outpatient review by the community team, she reported that she could manage insomnia after an MBT trial and trazodone 100mg at nighttime.

### Case 2

Mrs. B is a 49-year-old woman with a history of chronic pain, insomnia, depression (ICD.10: F 33.9), and anxiety (ICD.10: F 41.9) under our community’s care psychiatric team. She had a negative history of AUD. Her underlying neuropathy caused poor sleep and other psychological distress symptoms that were not responding to other painkillers (amitriptyline 10mg nocte) and routine hypnotic medication (zopiclone 7.5mg nocte). As her anxiety about pain and insomnia were mounting, she managed to have her PGN progressively increased in its dosage up to the maximum allowed by the national formulary of 600mg daily. Mrs. B tried stopping PGN by herself. However, she soon developed symptoms of withdrawal with rebound insomnia, anger, irritability, and anxiety.

At the beginning of the withdrawal stage, she presented with severe anxiety (GAD-7=19), severe clinical conditions (CGI-S=6), and moderate insomnia (ISI=21). After initially cross-tampering PGN with diazepam at 10mg three times daily, withdrawal symptoms subsided, hence reducing anxiety (GAD-7=3), improving clinical condition (CGI-S=1), and treating insomnia (ISI=7). DZ was then further reduced according to the cross-tapering scale. She was then discharged from the hospital after two weeks when she was prescribed regular mirtazapine 15mg once daily and promethazine 25-50mg nighttime when needed to help sleep. She was also referred to a clinical psychologist to start CBTI-MBT-SH. At the psychiatric community team review, 3-4 weeks after discharge, she no longer presented with symptoms of insomnia.

### Case 3

Miss C is a 50-years-old woman with a long history of polysubstance misuses (ICD10: F 19.0), anxiety (ICD.10: F 41.9), and borderline personality disorder (ICD-10, F 60.1) under the care of our community psychiatric team. She reported only occasional social alcohol consumption and no AUD. At hospital admission, she was severely ill (CGI-S=6), with severe anxiety (GAD-7=20), and severe insomnia (ISI=27). She presented with confusion, agitation, violence, assault of staff, and visual hallucinations. The urine test was negative for recreational drugs. She reported misusing PGN to deal with chronic anxiety, insomnia, and boosting other substances of abuse. She reached a daily use of PGN up to 900mg daily. The team started a short course of diazepam 5mg four times daily to manage PGN withdrawal symptoms with insomnia. In two weeks, she was successfully discharged home, and her test scores also improved (CGI-S=1: subtle pathology; GAD-7=6: mild anxiety; ISI=8 subthreshold insomnia). She was also referred to a clinical psychologist to start CBTI-MBT-SH while being in our community psychiatric team. She was started on mirtazapine 15mg nocte and promethazine 10 to 20mg nocte when required to help with her sleep.

## Discussion

The current study shows that when PGN is used for insomnia, persons might develop a dependence syndrome, while discontinuation causes withdrawal symptoms, initially producing rebound insomnia. Rapid discontinuation of PGN in the short term and an inpatient environment could bring some risk of rebound insomnia and risk of seizures^52,53^. Other authors’ advice is to taper the PGN steadily over one week while discontinuing it to reduce the risk of increased seizures, especially in people with a history of epilepsy^52,53^. Patients of other existing or previous substance use disorders, mainly opioid and multidrug patients, comprise the largest group at risk for gabapentinoid abuse, which can become lethal in overdose, especially in combination with opioids sedatives^53,54^.

The lesson learned is a caution note to all mental health professionals and family practitioners who should assess the real benefits and possible risks of using PGN for treatment-resistant insomnia. The clinical cases reported in the current study show a growing use of PGN in persons with problems with insomnia and anxiety who have tried other hypnotic medications with little effect. Although PGN was prescribed by clinicians to address stress and mild insomnia, patients soon started misusing it while developing a dependence syndrome with a rebound of insomnia at initial discontinuation. The behaviors linked to access additional PGN out of prescription are buying it online, getting it from drug dealers, piling PGN from different medical practitioners, or exaggerating symptoms of insomnia and anxiety to encourage clinicians to increase PGN dosage often above the amounts allowed by the national formulary. The authors of the current study also found that persons might take an extra dose of prescribed PGN to improve their sleep from their regular tablets.
